# Markers of Hepatic Insulin Clearance and Their Association With Steatosis in Hyperinsulinaemic Horses

**DOI:** 10.1111/jvim.70143

**Published:** 2025-06-06

**Authors:** Miranda Dosi, Laura Scott, Holly Payne, Jacqueline Poldy, John Keen, Bruce McGorum, Alexandra Malbon, Ruth Morgan

**Affiliations:** ^1^ Scotland Rural College Edinburgh UK; ^2^ Royal (Dick) School of Veterinary Studies and Roslin Institute University of Edinburgh Roslin UK

**Keywords:** CEACAM‐1, equine metabolic syndrome, insulin degrading enzyme, MASLD

## Abstract

**Background:**

Hyperinsulinaemia (HI) is an important feature of Equine Metabolic Syndrome (EMS). It has been suggested that reduced hepatic clearance of insulin contributes to HI, particularly in humans affected by metabolic dysfunction‐associated steatotic liver disease (MASLD).

**Hypothesis:**

In obese horses with HI, insulin clearance is impaired and associated with MASLD.

**Animals:**

Tissue samples were collected at post‐mortem from clinically well‐characterized horses with HI (*n* = 13; basal insulin > 20 mIU/l) and without HI (control; *n* = 20).

**Methods:**

Retrospective observational study. Molecular drivers of hepatic clearance (CAECAM‐1, an insulin chaperone protein and IDE‐Insulin Degrading Enzyme) were quantified by RT‐qPCR and activity, respectively, in liver tissue. Fixed liver sections stained with hematoxylin and eosin (H&E) were assigned a histological score by two blinded observers using an equine liver disease score and a human MASLD score. Triglyceride (TG) content in liver sections, serum liver enzymes, ACTH, basal insulin, and serum triglycerides were also measured.

**Results:**

IDE activity was 2.73 (IQR 4.00 activity/mg of protein) in HI horses and 2.18 (IQR 0.55) in controls (*p* = 0.07). IDE activity correlated negatively with insulin (rho = 0.561, *p* = 0.04) but not with liver or serum TG. CEACAM‐1 gene expression was higher in the HI group (2.09 ± 1.79 folds) than in controls (0.69 ± 0.62, *p* = 0.03). Liver disease and MASLD scores were no different between groups, whereas triglyceride liver content was higher in horses with HI (504.83 IQR 217.51 nmol/g) compared to controls (363.58 IQR 67.32 nmol/g, *p* = 0.04).

**Conclusions and Clinical Relevance:**

MASLD is not consistently present in HI horses, but CAECAM‐1 expression is higher.

**Abbreviations**
CEACAM‐1: carcinoembryonic antigen‐related cell adhesion molecule 1
EMS: equine metabolic syndrome
GGT: gamma glutamyl transferase
GLDH: glutamate dehydrogenase
H&E: hematoxylin‐eosin
HI: hyperinsulinaemia
ID: insulin dysregulation
IDE: insulin degrading enzyme
IR: insulin receptor
MAFLD: metabolic dysfunction‐associated fatty liver disease
NAFLD: non‐alcoholic fatty liver disease
TG: triglycerides

## 1 Introduction

Equine metabolic syndrome is a common endocrinopathy affecting horses, and insulin dysregulation (ID) is the critical feature [1]. In horses, ID can present as pre‐prandial hyperinsulinaemia (6–8 h with no access to food, HI), basal (fed) HI, or an exaggerated insulinaemic response to glucose. In humans and veterinary species, two basic mechanisms lead to chronic persistent hyperinsulinaemia: insulin hypersecretion and reduced hepatic insulin clearance [2, 3, 4, 5]. In physiological conditions, most of the insulin secreted by pancreatic β‐cells into the portal vein is degraded by the liver before reaching peripheral tissues [6]. Impaired insulin clearance, as opposed to insulin resistance and hypersecretion, has received less attention in the equine literature, and to date, there are only a few reports that document a role for this mechanism in equine ID [7, 8]. Insulin clearance is complex, but several proteins have been identified as critical for clearance. As the liver is responsible for metabolizing most of insulin, this study focused on two liver‐specific clearance proteins, namely Carcinoembryonic Antigen‐Related Cell Adhesion Molecule‐1 (CEACAM‐1) and insulin‐degrading enzyme (IDE). CEACAM‐1 is a well‐conserved transmembrane glycoprotein highly expressed on the sinusoidal surface membrane of hepatocytes [9]. This molecule mediates the endocytosis of the insulin‐insulin receptor complex into acidic endosomes for degradation [10]. In people with insulin resistance, hepatic CEACAM‐1 expression is reduced, and the amount of lipid accumulation in the liver is negatively and linearly correlated with CEACAM‐1 levels [11]. Moreover, liver‐specific CEACAM‐1 deletion in mice causes insulin resistance, HI, and increased hepatic lipid synthesis and accumulation [12, 13, 14]. IDE is considered the main protease responsible for the effective degradation of insulin in all insulin‐sensitive tissues [15]. Hepatic ablation of IDE in knock‐out mouse models produces HI and glucose intolerance [16, 17], whereas in obese humans, reduction of IDE activity aggravates the HI and facilitates the onset of type 2 diabetes mellitus [3, 6, 15].

In humans, persistent HI and insulin resistance are associated with a much‐increased risk of intra‐hepatocellular lipid deposition, resulting in steatosis or MASLD (Metabolic Dysfunction‐Associated Steatotic Liver Disease) [18]. The latter term highlights the presence of steatosis in association with features of metabolic syndrome rather than just the absence of alcohol consumption (non‐alcoholic fatty liver disease) [18, 19, 20]. The relationship between HI, steatosis, and insulin resistance is difficult to unpick, but it is thought that persistent HI and hepatic insulin resistance are both inciting causes and a result of MASLD, with a vicious cycle of pathology ensuing [21]. In horses, liver steatosis is only described in critically ill animals with severe HI secondary to a negative energy balance, but this does appear more common in horses with underlying endocrine disease [22, 23]. There is a paucity of data relating to hepatic lipid accumulation in horses with naturally occurring ID/HI in the absence of other systemic diseases.

In this retrospective observational study, we hypothesized that obese horses with fasting HI have decreased gene expression of CEACAM‐1 and decreased IDE activity. We also aimed to determine whether steatosis (MASLD) is present in horses with HI.

## 2 Materials and Methods

### 2.1 Animals

Serum and liver samples from 13 horses with HI (HI group) and 20 healthy controls were collected at euthanasia. The HI group was comprised of horses with a body condition score > 3.5, insulin > 20 mlU/L 8 h after food was withheld (Immulite 2000 xpi), and current/preceding history of laminitis [24]. ACTH was measured (Immulite 2000 xpi) to exclude the presence of Pituitary Pars Intermedia Dysfunction (PPID) according to published seasonally adjusted ranges [25]. The control group was comprised of lean horses without endocrine disease (insulin < 20 mIU/L and ACTH below seasonal ranges) that were euthanised for unrelated orthopedic disease and only included if they had no history or clinical signs of liver disease. Serum gamma glutamyl transferase (GGT) and glutamate dehydrogenase (GLDH) activities, triglyceride (TG), albumin, fasting glucose, and globulin concentrations were also measured. Homeostasis model assessment of insulin resistance (HOMA‐IR) was calculated as

$$\frac{\text{glucose}\times \text{insulin}}{450}$$

Where glucose is measured as mg/dl and insulin as mIU/l [26].

### 2.2 IDE Activity in Liver Tissue

Total protein concentration of each liver sample was determined using a Bradford Protein assay (Pierce Bradford Protein Assay Kit Thermofischer; Waltham, USA). IDE activity was determined using a fluorometric assay (Sensolyte 520 IDE Activity Assay, Anaspec, California, USA) [3]. Results are expressed as IDE activity/mg of protein.

### 2.3 CEACAM‐1 mRNA Quantification in Liver Tissue

Total RNA was extracted from frozen liver sections (20 mg) using the RNeasy Mini Kit (QIAGEN; Valencia, California, USA) according to the manufacturer's instruction. RNA quality was assessed using a Nanodrop Spectrometer. Quantitect Reverse Transcription Kit (QIAGEN; Valencia, USA) was used to synthesize cDNA from 500 ng of RNA. The CEACAM‐1 primers used were designed and validated ex novo. The housekeeping gene selected and validated was beta actin. Primers (Table 1) were designed using the National Center for Biotechnology Information (NCBI) primer Basic Local Alignment Search Tool (BLAST) according to the following criteria:

– Primers must span an exon‐exon junction;
– No complementarity with any other target;
– Similar GC% content and between 40% and 60%;
– Difference of melting temperature of maximum 2°C between forward and reverse sequences.

**TABLE 1:** Housekeeping gene (Beta Actin) and CAECAM‐1 primer sequences.

Gene	Accession	Primer sequence	Length	GC %	T	*R* ^2^	Efficiency%
Beta actin	NM_001081838.1	F: TACGGAGTACTTGCGCTCAG	20	52	54	0.93	107
		R: GGATGCAGAAGGAGATCACAG	21	55	54		
CEACAM‐1	XM_023651427.1	F: TGCATCATATAAGATAGGCCCAG	22	43	63.7	0.94	103
		R: AGTGAGAGTCCTCTTGTCCAGG	23	54	63.7		

*Note:* Both genes were validated on equine liver tissue. Rsq represents the coefficient of determination of the standard curve. Efficiency is expressed as a percentage and is calculated as −1 + 10^(−1/slope of standard curve)^.

Primer sequences and validation parameters are reported in Table 1.

Real‐time qPCR was carried out using the Agilent Mx3000 qPCR system (Agilent; Santa Clara, CA, USA), primer concentration determined to be optimal was 600 nM, and all reactions contained 10 ng of cDNA, 10 μl of SYBR Green, a dye and RNA‐free water (Applied biosystem, life technologies Corp; Carlsbad, CA, USA). The protocol consisted of a polymerase activation and cDNA denaturation at 95°C for 3 min, denaturation at 95°C for 15 s, then an annealing/extension process at 60°C for 20 s (40 cycles) and finally a melting phase at 65°C–95°C for 2–5 s. All samples were run in triplicate, and negative controls included. Data were recorded by MxPro qPCR software (Agilent; Santa Clara, CA, USA) exported to an excel sheet for further analysis, and outliers statistically excluded. Fold changes of gene expression were calculated with the ΔΔCt method and fold change of gene expression calculated as FC = 2^−ΔΔCt^.

### 2.4 Liver Histological Evaluation

Liver tissue was fixed in 10% formalin, paraffin‐embedded, 5 μm sections cut, and one stained with hematoxylin and eosin (H&E) and one with Masson's Trichrome to demonstrate collagen deposition (fibrosis). Each H&E stained section was scored using an equine liver disease scoring system [27] and a human MASLD scoring system [28, 29]. Scoring was carried out by two veterinary pathologists, blinded to the sample identity. Two sections from each horse were examined independently to ensure repeatability within and between observers. An in‐depth description and validation of the MASLD scale used is reported elsewhere [28, 29]. Steatosis was defined by the percentage of hepatocytes with lipid accumulation in the cytoplasm; the severity was Graded 0–3 (0 = less than 5%, Grade 1 = 5%–33%, Grade 2 > 33%, grade 3 > 66%). Ballooning was Graded from 0 to 2 (0 = normal hepatocytes with cuboidal shape and eosinophilic cytoplasm; 1 = presence of clusters of rounded hepatocytes with pale usually reticulated cytoplasm. Although hepatocyte shape is different, their size is quite similar to that of normal hepatocytes; 2 = as for Grade 1 with some enlarged hepatocytes, at least 2‐fold the diameter of normal cells). Lobular inflammation was defined as a focus of two or more inflammatory cells within the lobule. Foci were counted per 0.01mm^2^ field of view (FOV), with FN 22 at 200× magnification (0 = none; 1 = 1 or 2 foci per FOV; 2 ≥ 2 foci per FOV). Fibrosis was evaluated as a separate feature as part of the equine liver disease scoring system.

### 2.5 Hepatic TG Content

Triglycerides in frozen sections (100 mg of tissue) were quantified via a colorimetric assay used both in humans and veterinary species according to the manufacturer's instruction (AB65336; Abcam; Cambridge, UK) [30].

### 2.6 Statistical Analysis

Data were tested for normality (Shapiro–Wilk test); mean and standard deviation or median and interquartile range are presented accordingly. Inter‐group comparisons were made with parametric (t‐student) or non‐parametric (Mann–Whitney U) tests. For correlations, either Spearman (parametric variables) or Pearson's (non‐parametric variables) were used. Morphometric scores and histopathological scores being ordinal data were analyzed with non‐parametric tests. Non‐parametric tests were also used when continuous variables were not normally distributed. Significance was set at *p* < 0.05. Analyzes were carried out with SPSS 29 (IBM). Effect size was calculated as Cohen's d for parametric and *r* (*r* = test statistic/√*n*) and *η* ^2^ for non‐parametric variables.

## 3 Results

### 3.1 Horses

Table 2 summarizes the characteristics of the cohort enrolled in the study.

**TABLE 2:** Study sample characteristics.

Group	Age (years)	Sex	Breed	BCS/5	Insulin mIU/L	Glucose mg/dl	HOMA IR	History of laminitis
CONTROL *n* = 20	17, IQR 6	Geldings = 12, mares = 8	TB = 9, UK pony = 4, draught = 3, unknown = 2, KWPN = 1, ISH = 1	2*, IQR 1.1	2.00*, IQR 1.64	86*, IQR 16	0.79*, ±0.47	None
HI, *n* = 13	19, IQR 9	Geldings = 7, mares = 5, unknown sex = 1	UK ponies = 5, Cob = 3, Shetland = 2, Hanoverian = 1, Trakehner = 1, Unknown = 1	3.8*, IQR 1.0	38.4*, IQR 185.5	101*, IQR 25	31.73*, ±30.68	Yes = 9/13, no = 4/13

*Note:* Data are not normally distributed and are therefore reported as median and interquartile range (IQR). Homeostasis Model Assessment of Insulin Resistance (HOMA IR) was calculated as (glucose mg/dl × insulin mIU/L)/405 [26].

Abbreviations: ISH, irish sport horse; KWPN, dutch warmblood; TB, thoroughbred.

* *p* < 0.05.

### 3.2 Measures of Insulin Clearance

**FIGURE 1:**
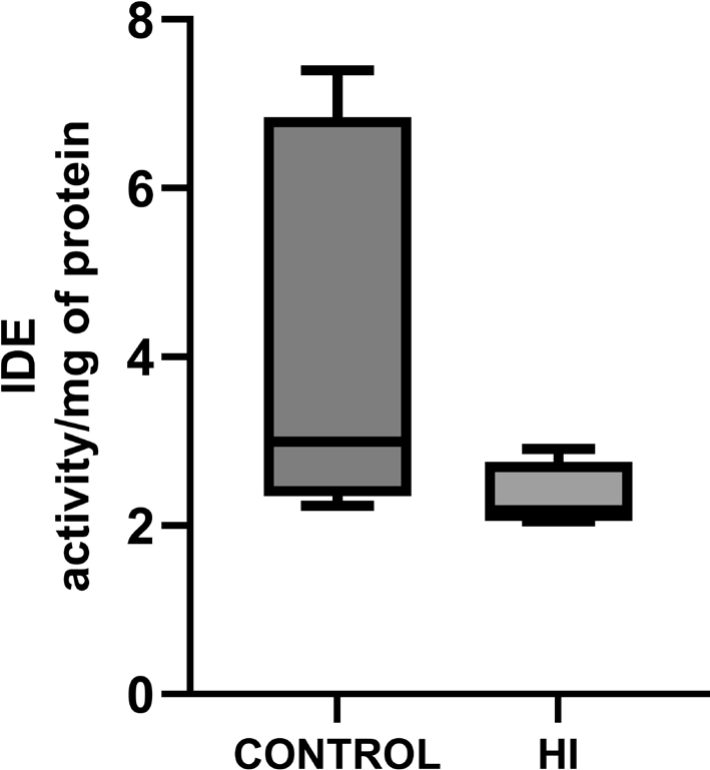
Graph showing IDE activity in control horses (3.00 IQR 4.04 activity/mg of protein; *n* = 8) compared to HI (2.18 IQR 0.40 activity/mg of protein, *n* = 5; *p* = 0.07).

**FIGURE 2:**
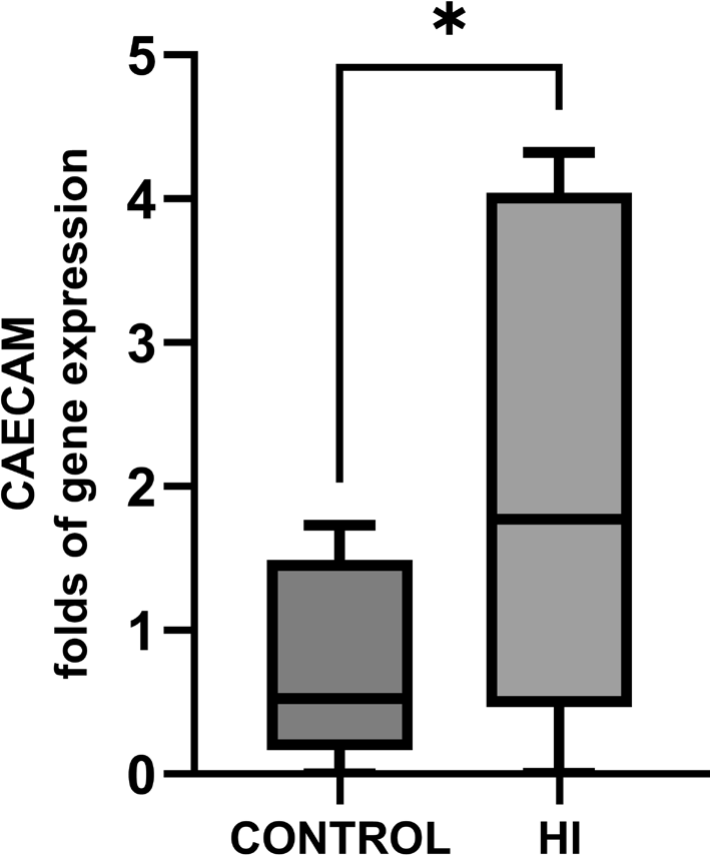
Graph showing difference in CEACAM‐1 in HI horses (1.86 SD ±1.81 folds, *n* = 9) compared to control horses (0.69 ± 0.62folds, *n* = 11, *p* = 0.03).

IDE activity analysis was carried out on 11 controls and 5 HI horses. IDE activity was 2.73 (activity/mg of protein‐IQR 4) and 2.18 (IQR 0.55) respectively in control and HI horses (*p* = 0.07 Figure 1). Although this difference was not statistically significant, the effect size was medium (*r* = 0.445, *η* ^2^ = 0.199), suggesting type‐2 error. Livers from 9 HI horses and 11 controls were available for CEACAM‐1 measurements. Horses in the control group had lower CEACAM‐1 mRNA expression (0.69 ± 0.62 mean fold gene expression) than HI horses (2.09 ± 1.79 mean fold gene expression; *p* = 0.03, Figure 2), although there was a large variability within the HI group. The effect size (Cohen's d −1.120; CI −2.090, −0.122) was large.

### 3.3 Measures of Steatosis

**FIGURE 3:**
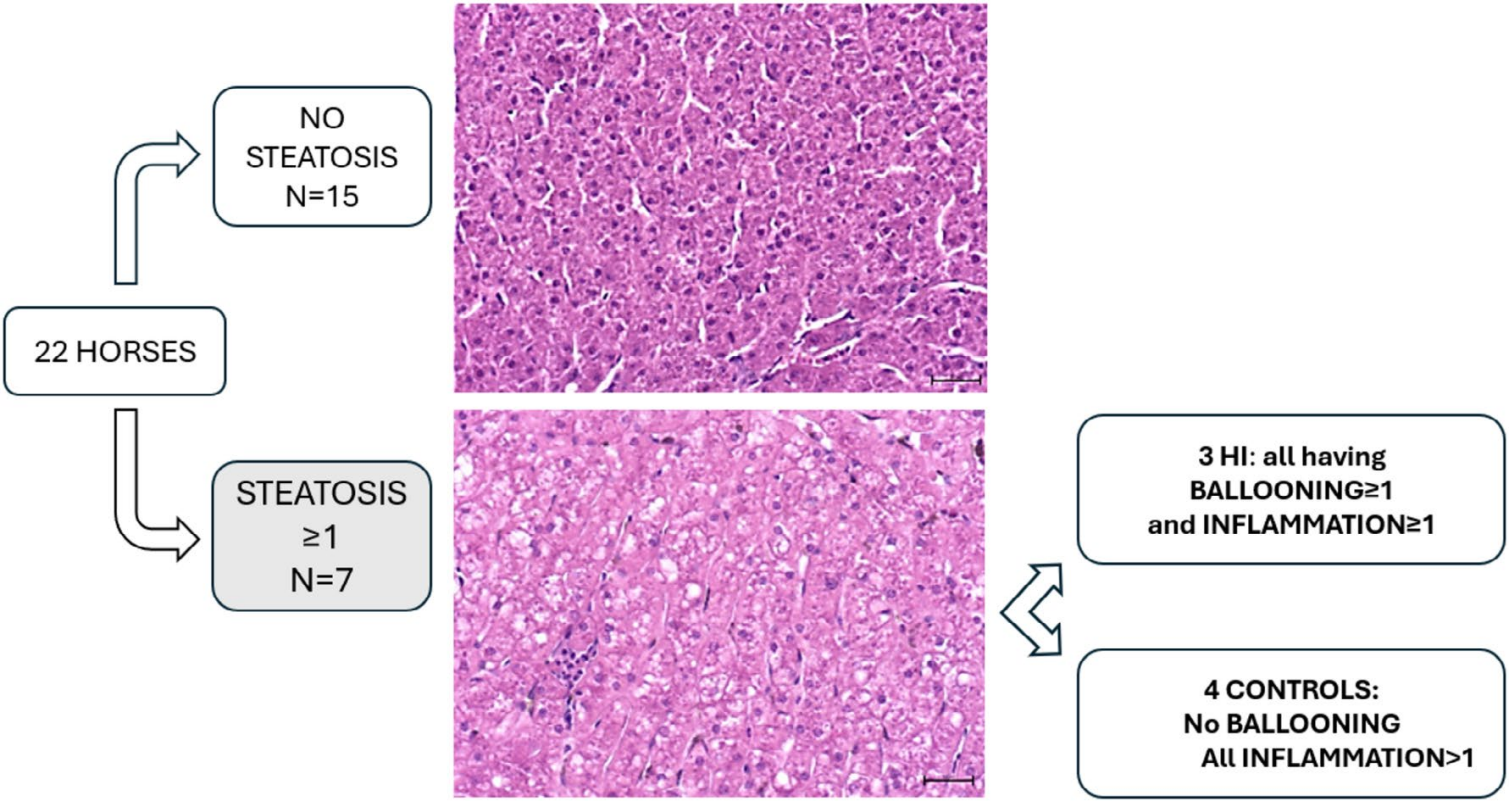
Graphic representation of the distribution of the study sample based on the MASLD scale. For simplicity, scores were reduced to binary (score = 0 for absence of the histopathological change, presence of changes all scores ≥ 1). For this analysis 14/22 controls and 8/13 HI horses' livers were available (magnification 20×, scale bar 50 μm).

Equine liver disease scores (controls 2 IQR 0 vs. HI 1 IQR 1.5) and MASLD scores (controls 2 IQR 2 vs. HI 3.5 IQR 3.25) were not different between groups. Inspecting Figure 3, which shows the distribution of the study sample based on the MASLD scoring system, some data stand out. Seven out of 22 horses had a steatosis score ≥ 1; of these, 4/7 were controls and 3/7 were HI horses. All horses with HI that had a steatosis score ≥ 1 also had ballooning, whereas in the control group, steatosis was not associated with ballooning. The vast majority of horses had evidence of inflammation (20/22 score ≥ 1), irrespective of their disease status.

**FIGURE 4:**
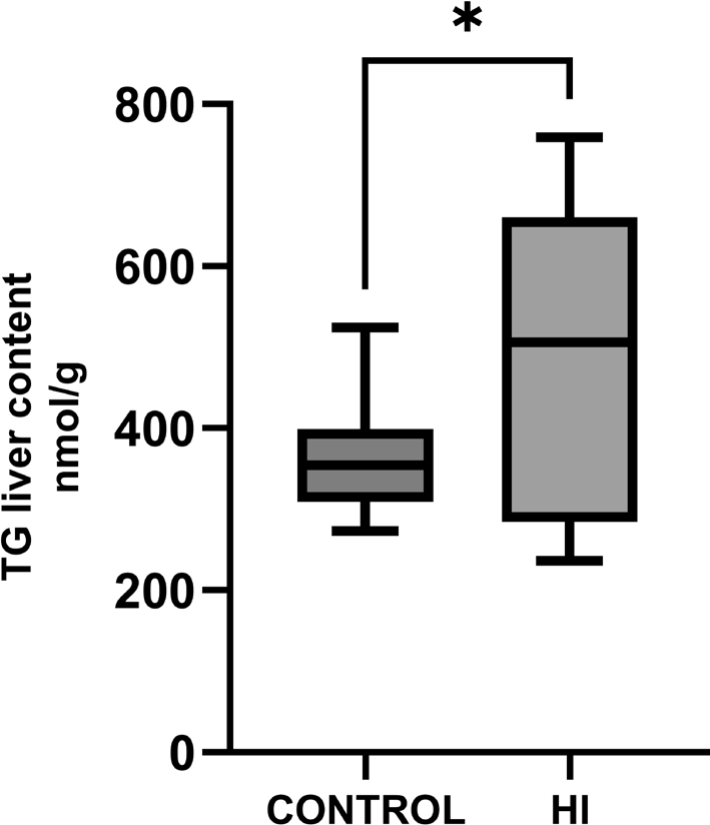
Graph comparing liver TG content in control horses (363.58 IQR 67.32 nmol/g, 480 *n* = 15) compared to HI horses (504.83 IQR 217.51 nmol/g, *n* = 8; *p* = 0.04).

When steatosis was assessed quantitatively by measurement of liver TG content (Figure 4), it was highly variable, being higher in HI horses (504.83 IQR 217.51 nmol/g) compared to controls (363.58 IQR 67.32 nmol/g, *p* = 0.04).

### 3.4 Serum Biochemistry

Serum GLDH was significantly higher in the HI (10.83 miUL/L IQR 9.63), although within laboratory reference ranges, than in control horses (2.8 IQR miUL/L 1.28; *p* = 0.004), whereas there was no difference in serum TG concentrations and GGT activity levels between the groups (Table 3). Upon further analysis, some breed‐associated differences were observed (see Section 3.6).

**TABLE 3:** Serum activities of GGT, GLDH, and concentration of TG in control HI horses.

	GGT IU/L	GLDH IU/L	Serum TG mg/dl
CONTROL	42.88 IQR 34.08	2.8** IQR 1.28	28.3 ±26.5
*N*	17	15	18
HI	52.36 IQR 30.84	10.83** IQR 9.63	45.13 ±9.73
*N*	11	12	13

*Note:* Only GLDH was statistically different between groups (** = *p* < 0.01). Due to the retrospective nature of the study, some samples were unavailable for testing; *N* indicates the tests performed for each group.

**TABLE 4:** Correlation Matrix: Multiple correlations were carried out and are expressed as Spearman (non‐parametric) or Pearson (parametric) coefficients.

	Steato‐sis score	MASLD score	Liver disease score	TG in liver tissue	IDE	CEACAM	Insulin	GGT	GLDH	TG in serum	Age	Breed
Steatosis score												
MASLD score												
Liver disease score												
TG in liver tissue												
IDE												
CEACAM												
Insulin												
GGT												
GLDH												
TG in serum												
Age												
Breed												

*Note:* A color code was given to represent the strength and direction of the correlation between variables, only statistically significant correlations were indicated by a colored box, non‐significant correlations were left white.
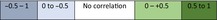

### 3.5 Multiple Correlation Matrix

Multiple correlation matrix analysis identified some statistically significant correlations between the variables measured (Table 4). Of note, measures of insulin clearance (CEACAM‐1 gene expression, IDE activity) did not correlate with measures of steatosis (MASLD scale, steatosis score, liver TG content). IDE activity, but not CEACAM‐1 expression, was negatively correlated with insulin (Spearman rho = −0.561, *p* = 0.03). There was a moderate but significant correlation between steatosis score and liver TG content (Spearman rho = 0.461, *p* = 0.05), and liver TG content correlated positively with insulin levels (Pearson *r* = 0.458, *p* = 0.04).

### 3.6 Breed Associated Differences

Further analysis of the data revealed that ponies, irrespective of their insulin levels, had significantly higher liver TG content (416.40 IQR 304.50 nmol/g; *n* = 9, 4 in control group and 5 in HI group) than TBs (349.52 IQR 105.98 nmol/g, *n* = 8; *p* = 0.04) which were all allocated in the control group. Thoroughbreds had lower serum GLDH activity (TBs 3 IQR 1.5 mIU/L) than ponies (6.5 IQR 24 mIU/l, *p* = 0.007), lower GGT activity (TB 24 IQR 1.5 vs. ponies 32.5 IQR 27.5, *p* = 0.016) and lower serum TG concentrations (TBs 0.26 ± 0.07 vs. ponies 0.63 ± 0.41 mmol/L, *p* = 0.04) than ponies.

## 4 Discussion

In this study we addressed the hypothesis that drivers of insulin clearance, such as CEACAM‐1 and IDE, would be reduced in obese horses and ponies with fasting HI and that these would present steatosis. Our findings confirm that CEACAM‐1 expression was altered in horses and ponies with HI, whereas IDE activity was variable. Steatosis was not consistently observed. On the other hand, HI horses had higher liver TG content than controls, and hepatic TG content was positively correlated with serum insulin level. Finally, we observed breed‐specific differences in serum and liver TG and liver enzymes that should be considered when investigating metabolic disease in horses.

### 4.1 Insulin Clearance

Insulin extraction rate can be directly calculated only by simultaneous catheterization of the portal and hepatic vein/central line. As this technique is impractical and achievable only in experimental settings, quantification of hepatic drivers of insulin clearance was carried out in this study. Insulin clearance is a complex process, requiring numerous receptor and mediator interactions, resulting in endocytosis of the hormone into hepatocytes. We chose two critical mediators of this process, IDE and CEACAM‐1, to determine if the molecular machinery of insulin clearance was altered in horses with HI. We identified a significant negative correlation between insulin level and IDE activity, suggesting that horses with higher serum insulin had lower IDE activity in their liver. Similarly, humans with type 2 diabetes have lower IDE activity, and knock‐out mice with specific hepatocyte IDE deletion develop hepatic insulin resistance and glucose intolerance when fed a hypercaloric diet [16]. On the other hand, in a similar study, the c‐peptide to insulin ratio was not different compared to the obese wild type, suggesting that IDE deletion might also affect hepatic insulin resistance and not just insulin clearance in mice [15]. Although a large variation was observed in the HI group, horses with HI had upregulated hepatocyte CEACAM‐1 expression, which is the opposite of that observed in mice and humans [11, 12, 13, 14]. CEACAM‐1 mRNA expression was measured via qPCR, which does not account for post‐transcriptional changes nor the phosphorylation necessary for CEACAM‐1 function. From our current observations, it could be hypothesized that the increased expression of CEACAM‐1 in the HI group compensated for the reduced IDE activity. Further studies to assess CEACAM‐1 at the protein level in larger groups could perhaps shed light on this.

### 4.2 Hepatic Steatosis

Obesity, hyperinsulinemia, and changes in insulin clearance in humans are strongly associated with the accumulation of lipid in the liver (MASLD), so we also investigated lipid accumulation in HI and control horses [31]. Current histological scoring systems used for the analysis of equine samples do not generally account for lipid accumulation; we therefore also applied a human MASLD scale [28, 29]. Hepatic steatosis in humans is diagnosed either by advanced imaging or liver biopsy and requires either liver tissue containing over 55 mg/g of TG or histopathological analysis that suggests ≥ 5% of the hepatocytes have accumulation of cytoplasmic lipids. We observed that the co‐presence of ballooning and steatosis was exclusive to the HI horses, whereas ballooning was absent in control horses with steatosis. Ballooning is a cytopathological abnormality characterized by the accumulation of small fat droplets, mitochondrial dysfunction, cellular oedema, endoplasmic reticulum expansion, and damage to intermediate filaments of the cytoskeleton, and has been associated with some clinical phenotypes, such as obesity and insulin resistance [32]. In our study sample, the prevalence of steatosis was too low to make any valid conclusions; therefore, the presence and meaning of these cytopathological changes in horses with ID warrant further investigation. Moreover, specific detection with a lipid stain, such as Oil Red O, would have better identified steatosis when this was more subtle had frozen sections been available because this cannot be performed on paraffin‐embedded tissue. We also incidentally identified a high prevalence of inflammatory foci in horses' livers, with most animals having an inflammation score ≥ 1. This has been observed in healthy horses [27] and it is possible that the equine liver has a normal resident population of immune cells that is not present in human liver parenchyma.

Despite the low prevalence of steatosis, we observed a positive correlation between the TG liver content and the histological steatosis scores, suggesting agreement between these methods of assessing steatosis. Horses with obvious lipid accumulation at histopathology had a lower TG liver content than humans diagnosed with MASLD [33]. This inconsistency could be explained by dietary factors, as horses have a low‐fat diet compared to humans and therefore are less prone to accumulate TG in their liver. Moreover, species‐specific differences in lipid metabolism might be a reasonable explanation for the comparatively lower TG content even in histologically steatotic livers, as in equids, de novo lipogenesis appears to occur mostly in the adipose rather than in the liver, unlike in humans [34]. Finally, lipid moieties other than TG may contribute to the lipid accumulation observed histologically in equine livers. TG are a relatively inert substance, and it is suggested that more hepatotoxic lipid compounds, such as free cholesterol, diacylglycerols, and sphingolipids, might contribute to the steatosis [35]. The latter are believed to directly interfere with insulin signaling and therefore participate in insulin resistance [35]. Of note, sphingolipid metabolism was studied in Icelandic ponies undergoing oral glucose tests and found that six types of sphingolipid were higher in the serum of ponies with greater insulin response [36]. Finally, the threshold for defining the degree of steatosis sufficient to cause liver damage or dysfunction in horses is unknown. Considering the significant species‐specific differences in lipid metabolism and diet composition between horses and humans, the adoption of the human threshold of clinically relevant steatosis might not be appropriate.

### 4.3 Breed‐Associated Differences

A confounding factor of this and most studies of HI is that of breed. For example, ponies are much more likely to develop EMS and HI than Thoroughbreds, and so it is difficult in clinical studies to unpick the differences driven by breed and those driven by HI. Despite our observation being limited by the small sample size, we found that ponies had higher liver TG concentrations than TB, irrespective of their serum insulin levels. Breed‐specific differences in liver fatty acid profiles and TG content have also been observed in Warmbloods (WB) and ponies fed identical diets [37]. In a diet‐induced model of obesity, it was found that despite an increase in basal insulin in both ponies and WB, only ponies developed a degree of steatosis [38]. In that study, similarly to our observations, ponies' serum GLDH activity remained persistently higher than those of WB. There is an increasing body of evidence suggesting that in pony breeds, many metabolic traits, such as serum TG, non‐esterified fatty acids, leptin, adiponectin, and insulin levels are genetically determined and have a moderate heritability [39].

## 5 Limitations

The retrospective nature, the small sample size, and unbalanced design are important limitations of this study. Our findings highlight how breed, independent of disease status, can affect clinical and histopathological variables. Although it is clear that an appropriately powered, case‐control, breed and age‐matched design could have yielded more conclusive results, this is hard to achieve using historical information and samples obtained at euthanasia from clinical cases.

Previous work on insulin clearance studied C‐peptide dynamics and enrolled horses with normal pre‐prandial insulin but inappropriate insulin and insulin: C‐peptide ratio during dynamic testing. These studies concluded that both pancreatic hypersecretion and reduced clearance participate in the exaggerated post‐stimulation insulin response [7, 8]. In the present study, C‐peptide was not measured, which would have been of interest, although reliable commercial assays are not readily available.

Our analysis did not include any marker of liver function, such as bilirubin or bile acids. The authors believe that as GGT and GLDH were not elevated in our study, it is unlikely that bile acids or bilirubin would be altered.

Finally, skeletal muscle and renal clearance of insulin were not addressed in this study.

## 6 Conclusion

This study suggests that hepatic insulin clearance and sensitivity could be altered in horses with persistent HI, and that local accumulation of lipids might participate in the pathogenesis of HI.

## Disclosure

Authors declare no off‐label use of antimicrobials.

## Ethics Statement

This study was approved by the University of Edinburgh Veterinary Ethics Committee (VERC 7014). Owners' consent was obtained at euthanasia. Authors declare human ethics approval was not needed.

## Conflicts of Interest

The authors declare no conflicts of interest.

## Supporting information


**Figure S1.** Liver section showing ballooning (arrows). Hepatocytes are enlarged, the cytoplasm is paler, and occasionally contains lipid droplets (H&E, 20×).
