# Supplementary figures and images for: Markers of Hepatic Insulin Clearance and Their Association With Steatosis in Hyperinsulinaemic Horses
Source: J Vet Intern Med. 2025 Jun 6;39(4):e70143. doi: 10.1111/jvim.70143 (PMC12143019; doi:10.1111/jvim.70143)

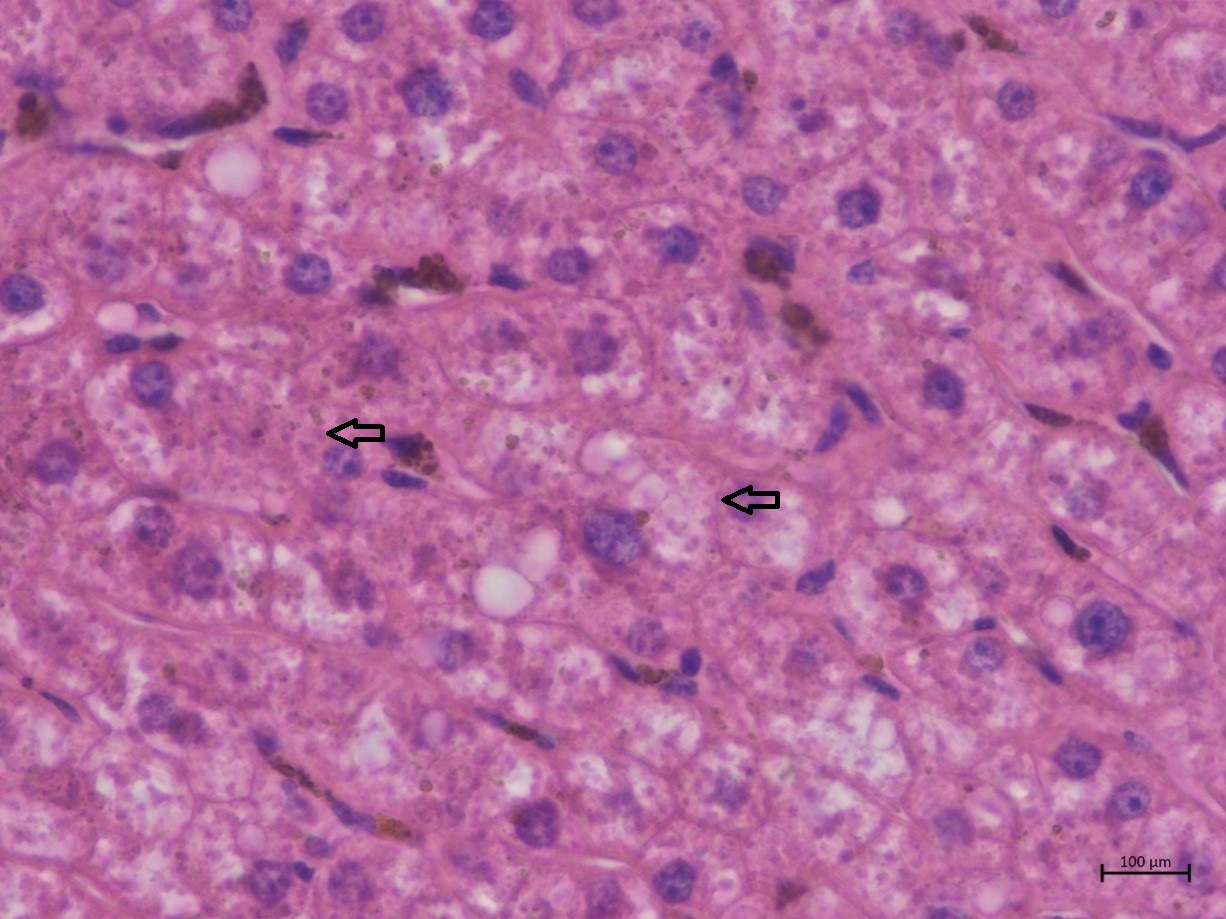

Supplement: Supplementary file 1 — Figure S1. Liver section showing ballooning (arrows). Hepatocytes are enlarged, the cytoplasm is paler, and occasionally contains lipid droplets (H&E, 20×). [file JVIM-39-e70143-s001.jpg]
